# Assessment of Knowledge, Attitude, and Practice Patterns in Pulmonary Arterial Hypertension among Cardiologists and Pulmonologists: Evidence from Turkey

**DOI:** 10.3390/medicina59101869

**Published:** 2023-10-20

**Authors:** Fatma Esra Günaydın, Erdal Belen, Sedat Altın, Ahmet Uğur Demir, Gülden Güven, Gündüz Durmuş

**Affiliations:** 1 Department of Immunology and Allergy, Ordu University Education and Research Hospital, Ordu 52200, Türkiye; 2 Department of Cardiology, Haseki Training and Research Hospital, University of Health Sciences, Istanbul 34265, Türkiye; belenerdal@gmail.com; 3 Department of Chest Diseases, Yedikule Chest Disease and Thoracic Surgery Education and Research Hospital, University of Health Sciences, Istanbul 34020, Türkiye; draltinsedat@gmail.com; 4 Department of Chest Diseases, School of Medicine, Hacettepe University, Ankara 06230, Türkiye; ademir68@gmail.com; 5 Department of Cardiology, Basaksehir Cam & Sakura City Hospital, Istanbul 34480, Türkiye; gldncz@hotmail.com; 6 Department of Cardiology, Dr. Siyami Ersek Thoracic and Cardiovascular Surgery Training and Research Hospital, Istanbul 34668, Türkiye; drgunduzdurmus@gmail.com

**Keywords:** pulmonary arterial hypertension, diagnosis, disease management, education, guideline adherence, questionnaire

## Abstract

*Background and Objectives*: Pulmonary arterial hypertension (PAH) is a rare chronic disease of the small pulmonary arteries that causes right heart failure and death. Accurate management of PAH is necessary to decrease morbidity and mortality. Understanding current practices and perspectives on PAH is important. For this purpose, we intended to determine physicians’ knowledge, attitudes, and practice patterns in adult pulmonary arterial hypertension (PAH) in Turkey. *Materials and Methods*: Between January and February 2022, an online questionnaire was sent via e-mail to all cardiologists and pulmonologists who were members of the Turkish Society of Cardiology (TSC) and the Turkish Thoracic Society (TTS). *Results*: A total of 200 physicians (122 pulmonologists and 78 cardiologists) responded to the questionnaire. Cardiologists were more frequently involved in the primary diagnosis and treatment of PAH than pulmonologists (37.2% vs. 23.8%, *p* = 0.042). More than half of the physicians had access to right heart catheterization. In mild/moderate PAH patients with a negative vasoreactivity test, the monotherapy option was most preferred (82.8%) and endothelin receptor antagonists (ERAs) were the most preferred group in these patients (73%). ERAs plus phosphodiesterase-5 inhibitors (PDE-5 INH) were the most preferred (69%) combination therapy, and prostacyclin analogues plus PDE-5 INH was preferred by only pulmonologists. *Conclusions*: Overall, clinical management of patients with PAH complied with guideline recommendations. Effective clinical management of PAH in specialized centers that having right heart catheterization achieve better outcomes.

## 1. Introduction

Pulmonary arterial hypertension (PAH) is a serious disease caused by high mean pulmonary arterial pressure (>20 mmHg) at rest due to elevated pulmonary vascular resistance (PVR) [1]. According to recent registry data, incidence and prevalence are ~6 and 48–55 cases/million adults, respectively [2]. Untreated PAH commonly causes right ventricular failure and death. Therefore, this diagnosis should be kept in mind for patients presenting with unexplained exertional dyspnea [3].

Treatment outcomes for PAH have significantly improved over the past decade. In addition, patients with PAH benefit most from a comprehensive treatment strategy and multidisciplinary care [3,4]. Endothelin receptor antagonists (ERAs), phosphodiesterase 5 inhibitors (PDE-5 inhibitors), prostacyclin analogues, and guanylate cyclase stimulants are the main medical treatment options. It is possible to improve the quality of life of PAH patients with an appropriate approach and ideal treatment management.

Physician questionnaires are useful for the assessment of diagnostic methods, disease management, and pharmacological effects in clinical practice, providing important information about factors that may affect the burden of PAH. In the literature, there are different perspectives on the diagnosis and treatment of PAH between countries [5,6,7,8,9]. There are very limited data on how clinicians diagnose and treat PAH in Turkey. Thus, the main objective of our study was to assess the knowledge, practices, and attitude towards PAH management in Turkey by conducting a physician-based study using a quantitative online questionnaire. In this study, we also aimed to compare clinical practice models, monotherapy, and combined therapy preferences in selected patients between pulmonologists and cardiologists.

## 2. Materials and Methods

### 2.1. Study Design and Participants

The universe of this study, which was carried out in Turkey between January and March 2022, consisted of cardiologists and pulmonologists with current memberships in the Turkish Society of Cardiology (TSC) and the Turkish Thoracic Society (TTS). At the time of this study, TSC and TTS had 3230 and 2872 members, respectively. Although membership in the relevant organizations is not compulsory, the majority of experts tend to be members of one of the relevant organizations. An online questionnaire was sent to all members of TSC and TTS on 26 January 2022. Four weeks later, the survey was shared once again on social media and was closed after a 10-week period. The prepared questionnaire protocol ensured the reliability of the data to be obtained, with full approval from doctors. In addition, the transparency and explanatory nature of the current survey allowed the initiating information to be maintained and the respondents could voluntarily withdraw from the study at any time. This study was approved by the regional ethics committee of Yedikule Hospital (identification no. 2021/122).

### 2.2. Questionnaire

The current questionnaire, which includes 31 questions, was prepared to evaluate demographic information, behavioral attitudes, and practices related to patient diagnosis, management, and compliance. We also added the questionnaire form we used in the study in English. The relevant form can be accessed in the “Supplementary Materials” section.

This questionnaire was developed based on previous studies as well as related sources such as the ESC and TTS guidelines [4,10]. It was carefully reviewed by two cardiologists and one pulmonologist who are experts in the treatment of pulmonary hypertension. They helped to improve the questionnaire’s quality and accuracy. The survey was presented in a multiple-choice format, offering participants the capacity to select multiple responses to specific questions. The survey was administered using a web-based form provided through Google’s questionnaire platform. Completion of the questionnaire required responding to all items included.

### 2.3. Statistical Analysis

Descriptive statistics were presented as numbers and percentages. Comparative analysis of study groups was analyzed using Pearson’s chi-square and Fisher’s exact tests. The statistical significance level of the *p*-value was accepted as <0.05. Statistical analyses were performed using SPSS v. 23.0 (International Business Machines Corp., Armonk, NY, USA).

## 3. Results

### 3.1. Demographics

A total of 200 doctors (122 pulmonologists and 78 cardiologists) participated in the present study. The response rates for TCS and TTS members were 2.4% and 4.2%, respectively. The majority of participating physicians, 96 (48%), were between the ages of 30 and 39. Of the respondents, 29 (14.5%) were residents, 66 (33%) were academics, and 105 (52.5%) were specialists. Of the clinicians participating, 41.5% worked at training and research hospitals, 29.5% worked at university hospitals, 20% worked at city and state hospitals, and 9% worked at private hospitals. Approximately 29% of respondents reported that they manage five to ten PAH patients each year, and 53.5% of physicians dedicate 5–9 min per examination. The demographic and characteristic features of the participants are shown in Table 1.

### 3.2. Availability of Diagnostic and Treatment Methods

More than 75% of the physicians had access to pulmonary function tests, echocardiography, and computed tomography. More than half of the physicians reported access to pulmonary rehabilitation (Table 1).

### 3.3. Management

Cardiologists were involved in the primary diagnosis and treatment of PAH more than pulmonologists (*p* = 0.042).

### 3.4. Treatment Preferences in PAH

**Figure 1:**
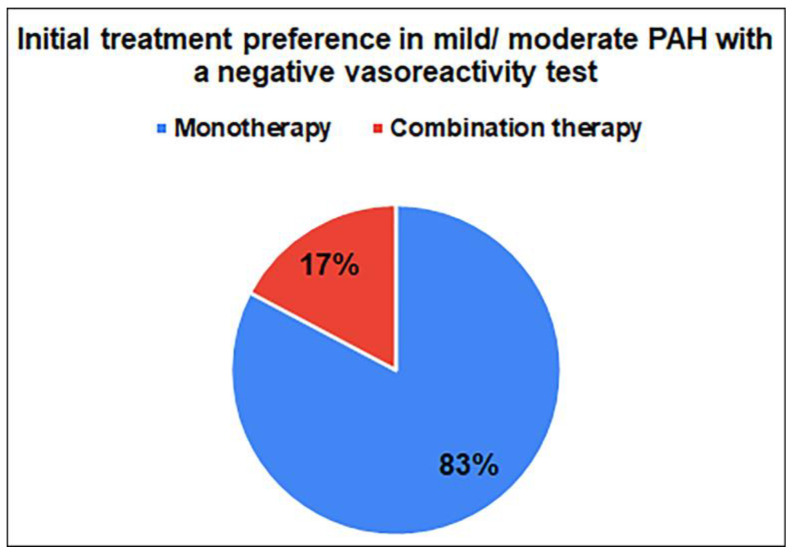
Initial treatment preferences of physicians for mild/moderate PAH diagnosed with a negative vasoreactivity test.

**Figure 2:**
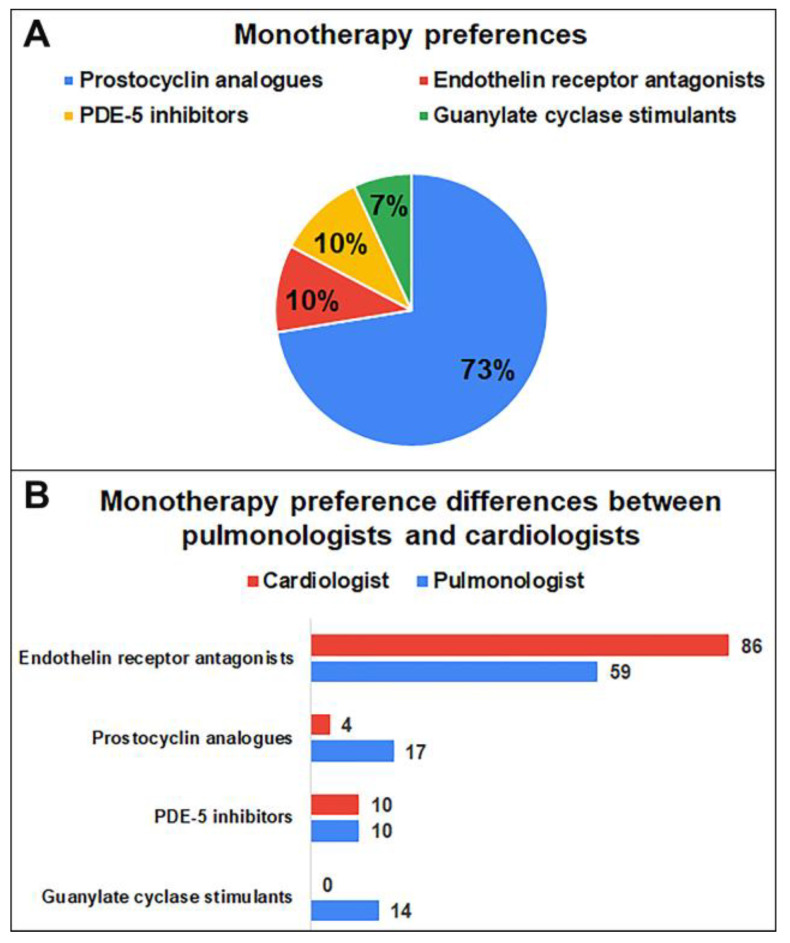
(**A**) Primary monotherapy preferences of physicians for PAH diagnosed with a negative vasoreactivity test (%). (**B**) Differences in primary monotherapy preferences among cardiologists and pulmonologists for PAH diagnosed with a negative vasoreactivity test (%).

The treatment received by patients with a diagnosis of pulmonary arterial hypertension before admission is usually combination therapy. Monotherapy was the preferred initial treatment in most (82.8%) patients diagnosed with mild/moderate PAH with a negative vasoreactivity test (Figure 1). ERAs were the monotherapy most preferred by doctors (73%), followed by prostocyclin analogues, PDE-5 inhibitors, and guanylate cyclase stimulants (Figure 2). Monotherapy preferences significantly differed between pulmonologists and cardiologists. Guanylate cyclase stimulants were preferred only by pulmonologists (*p* = 0.038).

**Figure 3:**
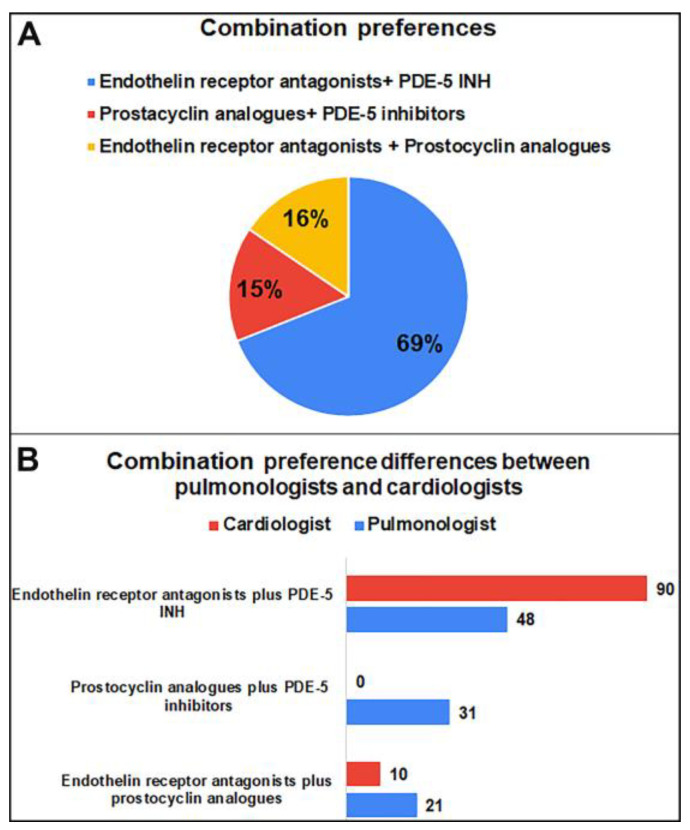
(**A**) Primary combination therapy preferences of physicians for PAH diagnosed with a negative vasoreactivity test (%). (**B**) Differences in primary combination therapy preferences among cardiologists and pulmonologists for PAH diagnosed with a negative vasoreactivity test (%).

ERAs plus PDE-5 INH were the most preferred (69%) combination therapy, followed by prostacyclin analogues plus PDE-5 inhibitors and ERAs plus prostocyclin analogues. Combination preferences significantly differed between pulmonologists and cardiologists in that prostacyclin analogues plus PDE-5 inhibitors were preferred only by pulmonologists (Figure 3) (*p* = 0.001).

### 3.5. Drug Choice and Evaluating Responses

A major determining factor that affected drug choice were guideline recommendations (96.6%), followed by side effects, effectiveness, prescribing advantages, and cost. Symptoms and 6 min walk tests were commonly used when evaluating responses to treatment. In some cases, 56.9% of physicians prescribed specific off-label drugs with the permission of the Ministry of Health.

### 3.6. Physician Beliefs on Patient Adherence and Symptom Control

Overall, 60.3% of the physicians believed that patients showed moderate adherence. They mostly believed that a major cause of non-adherence was lack of improvement of symptoms. Only 8.6% reported that patients with PAH had good symptom control and most of them (80%) were pulmonologists (*p* = 0.005). Reported good symptom control was not associated with specialty (*p* = 0.119).

## 4. Discussion

To the best of our knowledge, this is the first study to evaluate the adult pulmonary hypertension diagnosis, treatment, knowledge, attitude, and practice patterns among physicians working in Turkey. The key features and benefits of this study are information on the opinions of healthcare professionals, clinical management of patients with pulmonary arterial hypertension (PAH), as well as the needs and areas for improvement in the care of these patients in Turkey. Our study demonstrated that monotherapy was the most preferred (82.8%) monotherapy for patients with mild/moderate PAH diagnosed with a negative vasoreactivity test, and ERAs were the most preferred in this group (73%). ERAs plus PDE-5 INH were the most preferred (69%) combination therapy and prostacyclin analogues plus PDE-5 inhibitors were preferred only by pulmonologists.

Clinical assessment is a crucial part of evaluating patients with PAH, as it provides valuable information for determining disease severity, improvement, deterioration, or stability [4]. A considerable proportion of our participants reported managing more than five PAH patients annually. According to a study from Spain, the average number of PAH patients treated every year was found 39.5 ± 31.5 in their pulmonary hypertension unit [11]. Compared to the other study, our PAH patient number was lower because our study did not exclusively include pulmonary hypertension units; we preferred to include various centers that managed PAH to achieve a better understanding of real-life practice.

PAH symptoms are mostly connected with right ventricular (RV) dysfunction and are often associated with activity in the early stages of the disease [12]. The primary symptom is dyspnea which worsened by minor exertion.. The definitive diagnosis of PAH can be made by determining the mean pulmonary artery pressure, pulmonary capillary wedge pressure, and pulmonary vascular resistance obtained by RHC [13]. According to our questionnaire, more than half of the physicians had access to RHC in their hospital. In a study from Spain, RHC availability in one hospital was reported at 85% [11]. As a result of their international research, Preston et al. reported that the majority of patients did not undergo right heart catheterization for the diagnosis of PAH (not performed in the following numbers of cases: Argentina: 51%; United States: 21%; Europe: 7–21%; Japan: 19%) [13].

As for PAH treatment options, pulmonary angioplasty, septostomy, lung transplantation, etc., can mainly be performed in specialized units, according to European Society of Cardiology, European Respiratory Society, and Turkish Thoracic Society guidelines [4,10]. Lung transplantation especially remains a significant therapeutic option for patients with PAH resistant to optimal medical therapy, but it carries an intermediate-high or high risk of death [14]. It is also important to know how accessible lung transplantation is. In our study, the availability of lung transplantation in the participants’ city was 53%. In their study, Segovia-Cubero et al. found it to be at 65% [12].

For follow-ups on PAH patients’ symptoms, physical examination, the 6 min walk test (6 MWT), echocardiography, and RHC can be used. Of our participants, 79.3% used the 6 MWT and 48.3% preferred echocardiography for follow-up. Ryan et al. highlighted differences between clinicians practicing within and outside the United States (USA); clinicians practicing in the USA reported a similar tendency to utilize invasive cardiopulmonary hemodynamic assessment as a routine test during follow-up of PAH patients who had been prescribed vasodilator therapy. In addition, they reported that no significant difference was found between employed and contracted clinicians in the USA in terms of disagreement (dispute) scores regarding the use of the 6 min walk test and echocardiography to monitor the clinical progress of PAH patients in outpatient settings [15].

For the medical treatment of PAH, three routes that target pulmonary vasodilators are used, namely the endothelin pathway, the prostacyclin receptor effects on receptors, and the nitric oxide-cyclic guanosine monophosphate biological pathway. ERAs, PDE-5 inhibitors, prostacyclin analogues, and guanylate cyclase stimulants are the main treatment options. Combinations of pulmonary vasodilators that target several therapeutic pathways throughout the medical treatment outperformed monotherapy-based regimens in terms of survival and the amount of time until clinical deterioration [16,17,18,19,20]. As for treatment preferences, our participants mostly preferred monotherapy (82.8%) for patients with mild/moderate PAH diagnosed with a negative vasoreactivity test, and ERAs were the most preferred in this group. Similarly, in real-world data involving newly diagnosed USA patients with PAH, nearly 94% of patients commenced treatment with monotherapy and the most common treatment preference were PDE-5 inhibitors (70.0% of patients) [21]. Preston et al. found in their study that a significant proportion of patients were treated with PDE-5 inhibitors in Argentina and the USA, whereas in Europe, an equal proportion of patients were treated with PDE-5 inhibitors and ERAs [13]. In view of the increasing solid evidence for the efficacy of combination therapy as a treatment strategy in guidelines for the treatment of PAH, initial combination therapy may be considered at this risk level [4,10]. Approximately one-third of patients in Argentina, the United States, and Europe received combination therapy with PAH-specific drugs; it has been reported that 42% of patients in Japan were treated with combination therapy. However, a significantly greater proportion of patients in Japan reported receiving triple or more combination therapy compared to other regions [13]. Studer et al. reported that they found PDE-5 inhibitors plus ERAs to be the most common combination therapy in their study (53.9% of index combination regimens in the USA) [22]. Similarly, in our study, ERAs plus PDE-5 inhibitors were the most preferred (69%) combination.

Our study has several limitations. Firstly, the overall response rate was relatively low. Secondly, our questionnaire was designed based on the TTS and ESC/ERS guidelines, but it had not undergone validation. In this survey-based study, physicians reported their own behavior, so we can say that this may mean that it may not reflect clinical practice. Thirdly, we thought that differential diagnosis of PAH could be complicated and might require detailed information. Assessing and providing comorbidities to physicians could have enhanced—and broadened—differential diagnoses and treatment preferences for PAH patients. Since this was a first study, we did not use detailed descriptions and questions. We can add this information in a further study. In addition, we can say that participants who were unaware of the guidelines may have reported that their behavior may have met these standards, even if this was not the case. We think that a study based on a prescription database will yield more objective results regarding actual practice patterns. We think that it would be possible for them to be more compliant with current guidelines, since respondents who responded to the survey were the most interested in PAH.

## 5. Conclusions

In conclusion, our study revealed that physicians managing PAH implement the current ESC/ERS and TTS guideline recommendations for the diagnosis and treatment of pulmonary hypertension. Clinical management of PAH could have better outcomes in specialized centers equipped with most of the diagnostic methods and experienced physicians.

## Figures and Tables

**Table 1:** Characteristics of participants.

Characteristics	n (%)
**Age groups**	
20–29	25 (12.5)
30–39	96 (48)
40–49	56 (28)
50–59	16 (8)
60–69	7 (3.5)
**Specialty**	
Pulmonologist	122 (61)
Cardiologist	78 (39)
**Academic title**	
Residents	29 (14.5)
Specialists	105 (52.5)
Academics	66 (33)
**Workplace**	
University hospitals	59 (29.5)
Teaching and research hospitals	83 (41.5)
City and state hospitals	40 (20)
Private hospitals	18 (9)
**Number of PAH patients per year**	
Under 5	52 (26)
5–10	58 (29)
10–20	43 (21.5)
20+	47 (23.5)
**Patient examination time (minutes)**	
Under 5	42 (21)
5–9	107 (53.5)
10–14	25 (12.5)
15–19	13 (6.5)
20+	13 (6.5)
**Availability of diagnostic methods**	
Pulmonary function test	156 (78)
Diffusing capacity for carbon monoxide	60 (30)
Echocardiography	183 (91.5)
Computed tomography	167 (83.5)
Right heart catheterization	107 (53.5)
**Availability of treatment methods**	
Pulmonary rehabilitation	109 (54.5)
Endarterectomy	79 (39.5)
Lung transplantation	77 (38.5)

## Data Availability

The data and materials used in this study are available upon reasonable request from the corresponding author. Restrictions may apply to the availability of certain data sets due to privacy or ethical restrictions.

## Supplementary Materials

The following supporting information can be downloaded at: https://www.mdpi.com/article/10.3390/medicina59101869/s1, Supplementary data: Questionnaire used in this study.
