# Supplementary material for: Assessment of Knowledge, Attitude, and Practice Patterns in Pulmonary Arterial Hypertension among Cardiologists and Pulmonologists: Evidence from Turkey
Source: Medicina (Kaunas). 2023 Oct 20;59(10):1869. doi: 10.3390/medicina59101869 (PMC10608633; doi:10.3390/medicina59101869)
Supplement: Supplementary file 1 [file medicina-59-01869-s001.zip › medicina-2629278-supplementary.pdf]

Evaluation of physicians' clinical approaches and practices in the diagnosis and treatment of patients with pulmonary arterial hypertension

Dear professors and colleagues,

In our study titled "Evaluation of clinical approaches and practices of physicians in the diagnosis and treatment of patients with pulmonary arterial hypertension (PAH) ", we aimed to evaluate diagnostic methods, treatment preferences and practical approaches to PAH in our country.

The duration of the survey is 5-7 minutes.

Thank you very much for your support.

Best regards

Dr Aaaa Aaaaaa

Dr Bbbb Bbbbb

Dr Cccc Ccccccc

Dr Dddd Dddddd

Dr Eeee Eeeeeee

Dr Ffff Ffffffff

Questionnaire form

1. Age

- 20-29 age
- 30-39 age
- 40-49 age
- 50-59 age
- 60-69 age
- 70 age and older

2. Gender

- Female
- Male

3. City you work in

…………

4. Workplace

- University hospitals
- Education and research hospitals
- City and State hospitals
- Private hospitals

5. Speciality

- Cardiology
- Pulmonology

6. Academic title

- Residents
- Specialists
- Academicians

7. How many minutes you have for per inspection? (Select one)

- Less than 5 minutes
- 5-9 minutes
- 10-14 minutes
- 15-19 minutes
- 20+ minutes

8. Although there is no specific symptom in pulmonary hypertension, the most suspicious symptom/symptoms in your opinion (You can tick more than one option)

- Shortness of breath
- Palpitations
- Fatigue
- Cough
- Chest pain
- Syncope

9. In your outpatient practice, when categorizing patients with pulmonary hypertension (PH) based on their underlying causes, please arrange them in order of frequency

	1 st	2 nd	3 rd
Pulmonary arterial hypertension			
Pulmonary hypertension with left heart disease			
Pulmonary hypertension secondary to lung diseases and/or chronic hypoxaemia			

10. When there is a suspicion of pulmonary hypertension, which test or tests are readily available for you to order? (You can choose multiple options)

- Pulmonary function tests
- Diffusing Capacity of Carbon Monoxide
- Echocardiography
- Computerized tomography

11. For patients with echocardiographic findings suggesting intermediate to high likelihood of pulmonary hypertension, do you order ventilation/perfusion scintigraphy to evaluate chronic thromboembolic pulmonary hypertension (CTEPH)?"

- Always
- Sometimes
- Never

12. Is right heart catheterization available at your institution?

- Yes
- No

13. Is there a unit/center in your province where you can refer patients for the pulmonary rehabilitation of patients with pulmonary hypertension (PH)?

- Yes
- No

14. Is there a facility in your working city where you can refer patients with pulmonary hypertension who require lung transplantation?

- Yes
- No

15. In the city where you work, is there a surgical center that you can refer patients diagnosed with CTEPH for the endarterectomy?

- Yes
- No

16. Your approach to your PAH patients who want to get pregnant (Select one)

- I recommend never getting pregnant
- I suggest getting pregnant by explaining the risks

17. Patients with the worst prognosis (Select one)

- Pulmonary arterial hypertension
- Pulmonary hypertension with left heart disease
- Pulmonary hypertension secondary to lung diseases and/or chronic hypoxaemia

18. In your opinion, should PAH screenings be conducted in high-risk populations?

- Yes
- No

19. Are patients suspected of having PAH due to connective tissue diseases, HIV, drug-related conditions, or similar illnesses referred to you from other specialties?"

- Yes
- No

20. How many patients do you encounter with PAH in your daily practice yearly?

- Less than 5
- 5-9
- 10-14
- 15-19
- 20+

21.The treatment received by patients diagnosed with pulmonary arterial hypertension is usually (Select one)

- Monotherapy
- Combination therapy

22. Are you involved in the primary treatment and follow-up of patients with pulmonary arterial hypertension?

- Yes
- No

23. In a mild/moderate risk patient with a negative vasoreactivity test and diagnosed with pulmonary arterial hypertension (PAH), your choice of starting treatment is usually

- Monotherapy
- Combination therapy

24. Patients with negative vasoreactivity test PAH , what is your primary preferred monotherapy choice?

- Prostocycline analogues
- Endothelin receptor antagonists
- PDE- 5 inhibitors
- Guanylate cyclase inhibitors
- IP receptor agonists

25. Patients with negative vasoreactivity test PAH , what is your primary preferred combination therapy choice?

- Endothelin receptor antagonists plus PDE- 5 inhibitors
- Prostocycline analogues plus PDE- 5 inhibitors
- Endothelin receptor antagonists plus Prostocycline analogues

26. What is the most crucial factor or factors influencing your selection of specific medication? (You can choose multiple options)

- Ease of prescribing
- Guide recommendation
- Effectivity
- Price

27. Your most important criteria for evaluating response to treatment (You can tick more than one option)

- Symptom
- Physical examination
- 6 Minute walk Test
- Echocardiography
- Right heart catheterization

28. Do you ever have to prescribe original medications off-label for PAH?

- Yes
- No

29. How is drug compliance in patients who have started PAH treatment?

- Good
- Average
- Bad

30.Percentage of symptom control with treatment in PAH patients;

- 80 and above
- 60- 79
- 40-59
- 20-39
- Less than 20

31. In your experience, what do you think are the major factors for patients' non-adherence to the prescribed treatment? (Can select more than one)

- Side effects of medication
- No improvement in symptoms
- Knowledge of poor prognosis
- Challenges in accessing medications, associated tests, and medical care
